# Advancement in Understanding Glaucoma: A Comprehensive Review

**DOI:** 10.7759/cureus.46254

**Published:** 2023-09-30

**Authors:** Azeem I Saifi, Prachee Nagrale, Khizer K Ansari, Iram Saifi, Sharad Chaurasia

**Affiliations:** 1 Medicine, Jawaharlal Nehru Medical College, Datta Meghe Institute of Higher Education & Research, Wardha, IND; 2 Ophthalmology, Jawaharlal Nehru Medical College, Datta Meghe Institute of Higher Education & Research, Wardha, IND; 3 Medicine and Surgery, Jawaharlal Nehru Medical College, Datta Meghe Institute of Higher Education & Research, Wardha, IND; 4 Radiology, Jawaharlal Nehru Medical College, Datta Meghe Institute of Higher Education & Research, Wardha, IND

**Keywords:** aqueous humour, intraocular pressure, advance treatment, glaucoma, genetic

## Abstract

Glaucoma, a silent thief of sight, remains a significant cause of irreversible blindness due to a substantial number of undiagnosed and untreated cases. To combat this insidious disease effectively, a multifaceted approach is imperative. Early detection is paramount in the battle against glaucoma. Patient history, including family history, plays a pivotal role in identifying those at risk. A comprehensive understanding of a patient's genetic predisposition can significantly enhance the accuracy of diagnosis and detection of suspicious cases. Treatment options include prescription eye drops, oral medicines, laser treatment, surgery, or a combination of approaches. Trabeculectomy involves the surgical creation of an aqueous humor drainage channel, while laser trabeculoplasty enhances aqueous outflow by modifying the trabecular meshwork. However, these procedures pose certain risks and complications. Exploration of alternative treatments with lower risks is underway. These innovative approaches hold promise in reducing the burdens associated with conventional treatments such as trabeculectomy. However, the effectiveness of these alternatives in the long term remains a subject of ongoing research. Neuroprotective drugs have also been in development to halt the progression of glaucoma. However, their success remains uncertain due to challenges, such as a lack of understanding of the underlying mechanisms, scarcity of suitable drugs, and regulatory hurdles in gaining approval. In essence, the overarching goal of glaucoma therapy is to reduce intraocular pressure through various means - medications, laser procedures, or innovative methods. The aim is to slow down the disease's progression, thereby preserving vision and improving the patient's quality of life. In conclusion, addressing the challenge of glaucoma requires a comprehensive approach encompassing early detection, innovative treatments, and ongoing research into potential cures. Only through concerted efforts can we hope to reduce the impact of this sight-stealing disease on individuals and society as a whole.

## Introduction and background

Glaucoma is characterised by the progressive loss of retinal ganglion cells (RGC) and particular alterations in the neuro-retinal rim tissue situated in the optic nerve head (ONH), resulting in a narrowing of the visual field (VF) [1]. It is one of several eye disorders that are the leading cause of permanent blindness globally [2]. The risk of getting glaucoma is not only high, but there is also worry about untreated instances, which can result in irreversible visual loss [1]. For example, a study of 5,000 urban Greek people aged 59 and up indicated that 57.1% of glaucoma cases went undetected [3]. Similarly, in a study of mostly white Australians (3,654 participants, 90.2% of whom were 60 or older and 24% of whom were 80 or older), 3.0% had primary open-angle glaucoma (POAG), while 51% had previously gone untreated [4]. Because of the high number of undiagnosed glaucoma cases, research done in hospitals or specialised clinics may introduce bias by focusing on certain referred individuals, failing to reflect the larger glaucoma community. Population-based studies are required to identify risk variables related to the development of glaucoma [5].

The prevalence or incidence of glaucoma in such investigations is determined by the extent of the examination technique used. A thorough patient history can help identify those who are at risk of developing glaucoma even before any glaucomatous changes appear. Furthermore, historical data can help in making educated treatment decisions, defining follow-up plans, and estimating the pace of glaucoma development [6]. Individuals might be classified as healthy, glaucoma suspects, or those with glaucomatous pathology based on their patient history and objective tests. Furthermore, in demographic research and clinical settings, family history can improve the accuracy of glaucoma diagnosis [7]. In fact, according to research done in a general medical outpatient context, an accurate diagnosis was determined in 82.5% of patients based on the medical history supplied [8]. While objective data from instrument-based tests are routinely used in glaucoma research to generate diagnostic classifications, patient history and other clinical findings are usually disregarded [9-13]. Some research [6,14,15] concentrates primarily on family history rather than a thorough patient history. Relying primarily on glaucomatous pathology findings may restrict the discovery of glaucoma suspects. While increased technology in glaucoma detection tools is useful, it is critical not to disregard information available from a patient's history. For example, Miki et al. found that the rate of retinal nerve fibre layer (RNFL) loss over time might assist in identifying individuals at risk of developing VF loss, implying that OCT alone could be effective as an evaluation tool [16]. However, given the potential benefits of thorough patient history, it begs the issue of whether more people at risk of lower RNFL thickness and VF loss may have been recognised. Combining RNFL thickness analysis with visual function tests can enhance glaucoma detection [17].

The objective of this study is to reevaluate the possible use of a patient's medical history in the detection of suspicious cases and diagnosis of glaucoma. The identification of glaucoma suspects mainly relies on the patient's medical history when there is no objective proof of the disease. In order to reduce the danger of unnoticed disease development, it is essential to check glaucoma patients more often [18]. Adopting dynamic and personalised testing methods has been proven to improve the efficiency of spotting the onset of OAG and shorten diagnostic times in contrast to fixed yearly monitoring intervals [18]. Monitoring programmes should ideally be tailored based on each patient's unique risk factors for the onset or advancement of glaucoma. Forecasting models may be more accurate if they take into account important data from a patient's medical history. This assessment focuses on certain historical facts that may be useful in identifying diagnostic subtypes and directing individualised therapy and follow-up strategies in accordance with the person's risk profile and anticipated pace of illness development.

## Review

Methodology

**Figure 1:**
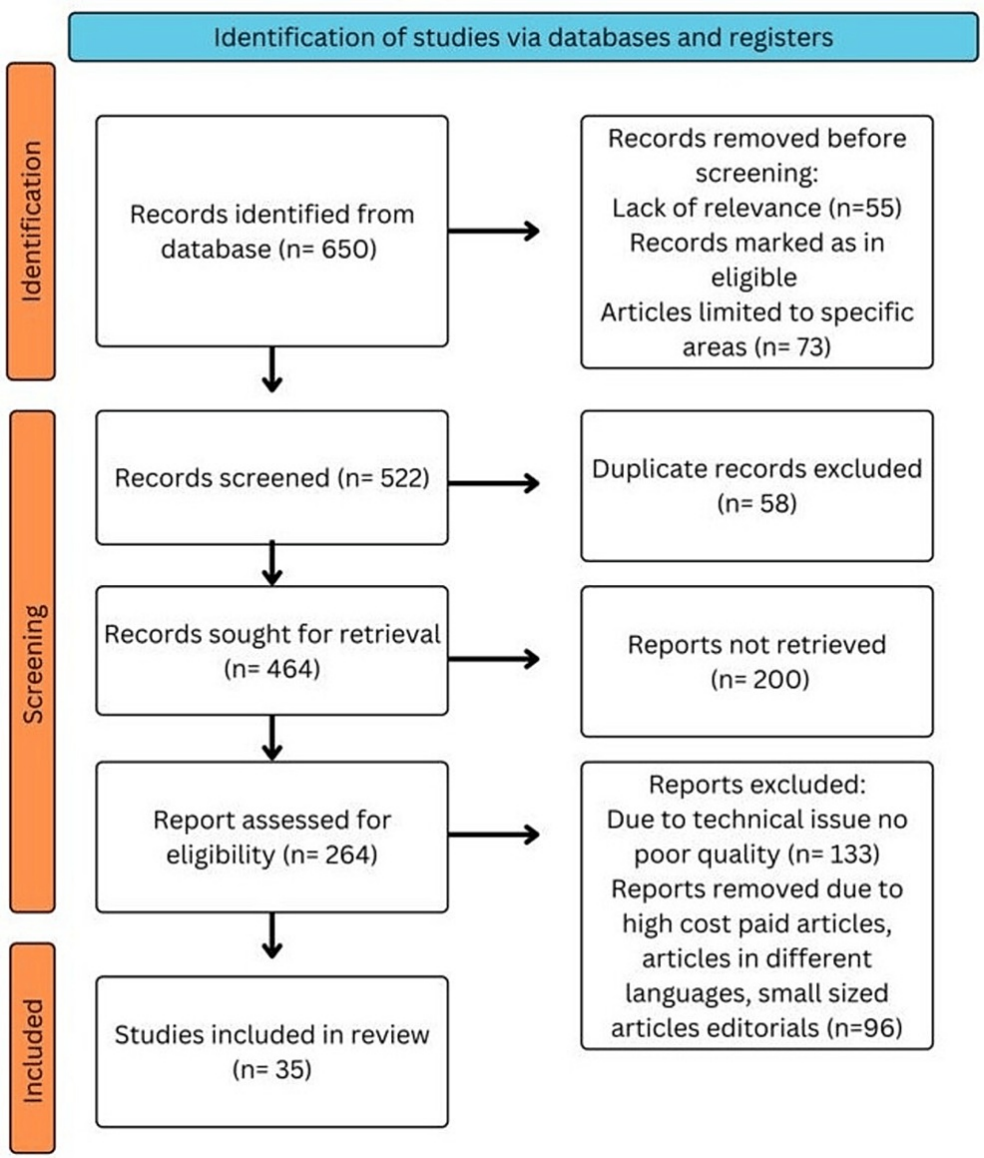
PRISMA Methodology PRISMA: Preferred Reporting Items for Systematic Reviews and Meta-Analyses

We comprehensively searched the electronic databases PubMed, MEDLINE, Embase, Google Scholar, and ResearchGate, and a search of the English-language literature was done. It was also the subject of a different search. The query terms were "Ocular hypertension" OR "Intraocular pressure"; "Open-angle glaucoma" OR "Angle-closure glaucoma; "development," OR "progression”; “Medical management of glaucoma” OR “IOP-lowering medications”; "Primary glaucoma” OR "Secondary glaucoma.” The articles in this review meet the following requirements: studies conducted exclusively on glaucoma, drug-resistant glaucoma, and new treatment interventions are considered. Studies conducted in English over the preceding 10 years are also included. Figure 1 highlights the Preferred Reporting Items for Systematic Reviews and Meta-Analyses (PRISMA) method used in the research methodology.

Risk factor 

Age and Frailty

It is generally known that, as people age, their chance of acquiring glaucoma rises. Therefore, macular degeneration, vascular disease, and obstructive sleep apnea are among the age-related diseases that frequently coexist with glaucoma. It is vital to remember, nevertheless, that most age-related illnesses do not directly correlate to these correlations as causes. Frailty, on the other hand, is a condition of vulnerability that puts people at a higher risk of early ageing, disability, and death. Frailty is characterized by the buildup of health problems, which can include illnesses such as diabetes, migraine, obstructive sleep apnea syndrome, cataracts, glaucoma, and drug use. Similar to this, the frequency of numerous health diseases tends to rise with frailty, which might account for some people having both of these ailments at the same time. It is important to remember that a fragile person may have a higher chance of acquiring glaucoma even when they are younger [15].

Gender

A risk factor for the onset of POAG in the ocular hypertension treatment (OHT) study was univariate analysis, which revealed that males may be more likely to develop POAG. In addition, a Bayesian meta-analysis revealed that men are more likely than women to develop open-angle glaucoma (OAG), albeit this connection may vary depending on the precise definition of glaucoma utilized. It is crucial to remember, though, that, depending on the kind of glaucoma under consideration, the effect of gender on the disease might change. For instance, a study of the literature revealed that women are more likely than males to develop angle-closure glaucoma (ACG), but OAG has no obvious gender preference. These results need to be understood in light of the populations under investigation. Women are also more likely than males to acquire glaucoma and endure glaucoma-related visual loss since they typically live longer [15].

Myopia

Myopia is a substantial risk factor for the development of glaucoma, according to studies. The higher incidence of myopia among Asian patients may help explain why this demographic has a higher prevalence of glaucoma. Furthermore, in some age groups, excessive myopia and increasing axial length have been identified as significant risk factors for glaucoma. These data suggest that the degree of myopia is related to the likelihood of acquiring and advancing glaucoma [15].

Migraine

Normal-tension glaucoma (NTG) and migraine have been linked, suggesting that both disorders may have the same vascular etiology. Additionally, among those between the ages of 70 and 79, a statistically significant connection between OAG and migraine was discovered [15].

Pigmentary Dispersion Syndrome

Myopic people are the main victims of pigmentary glaucoma, especially young people with pigment dispersion syndrome. In instances involving pigment dispersion syndrome, a number of variables have been identified as possible risk factors that may contribute to the development and seriousness of glaucoma. These variables include male gender, African American race, severe myopia, and the presence of Krukenberg spindles [15].

Pseudo-Exfoliation Syndrome

Pseudo-exfoliation syndrome is a typical fibrotic matrix disorder that commonly develops with ageing and is known for its link to an increased risk of glaucoma [15].

Diabetes

In all six case-control studies analyzed in the meta-analysis by Zhou et al., diabetes was found as a risk factor for POAG with a mean odds ratio larger than one. However, the odds ratio for the sixth experiment was 0.61. Moreover, five of the six population-based cohort studies that were a part of the analysis discovered a substantial relationship between diabetes mellitus and POAG. Diabetes patients are thought to have a higher prevalence of POAG because of their greater sensitivity to intraocular pressure (IOP) and increased risk of neuronal damage from hyperglycemia. Based on a comparison of 80 patients with NTG and 4015 control patients in a Korean population, a higher proportion of fasting capillary glucose levels above 200 mg/dL was found to be a risk factor for OAG in both univariate and multivariate analyses. It is crucial to remember that there is still disagreement on the link between diabetes and glaucoma [15].

Smoking

**Figure 2:**
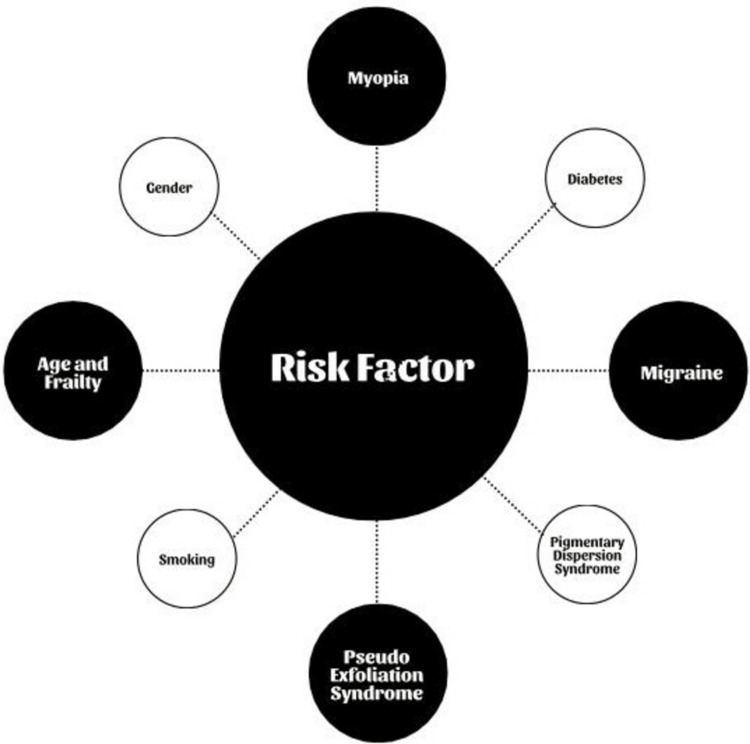
Risk Factors [Image credits: Azeem I. Saifi]

The outcomes of investigations looking into the link between glaucoma and smoking have been inconsistent, generating contradicting results. It has been postulated that, when people with a genetic predisposition are exposed to environmental variables including smoking, corticosteroid treatment, and diabetes, glaucoma may develop at a younger age. Furthermore, research suggests that the incidence of glaucoma among smokers is greater in men [15]. All the risk factors are mentioned together in Figure 2.

POAG

Glaucoma's specific underlying processes are still not completely understood. IOP and the death of retinal ganglion cells do, however, appear to be related. The equilibrium between the ciliary body's generation of aqueous humor and its drainage through the trabecular meshwork and uveoscleral outflow route affects IOP. ACG patients frequently have an obstruction in the drainage routes, whereas those with OAG generally exhibit greater resistance to aqueous outflow through the trabecular meshwork. Elevated IOP can put the posterior structures of the eye under mechanical stress and strain, especially the lamina cribrosa and the tissues around it. Optic nerve fibres (retinal ganglion cell axons) escape the pressurized eye through the lamina cribrosa, which is also its weakest point. Elevated IOP can cause the lamina cribrosa to compress, deform, and remodel, which can cause mechanical injury to the axons and interference with axonal transmission [19]. These mechanisms have a role in both the glaucoma-specific optic nerve injury and the gradual loss of retinal ganglion cells. Important trophic components cannot be supplied retrogradely from the brainstem target (relay neurons of the lateral geniculate nucleus) to the retinal ganglion cells because glaucoma disrupts axonal transport at the lamina cribrosa level. In experimental research, orthograde and retrograde axonal transmission were found to be reduced in this area in animals with induced ocular hypertension, including cats and monkeys. Ultrastructural changes in optic nerve fibres, including vesicle accumulation and dis-organisation of microtubules and neurofilaments, have been seen in post-mortem human eyes with glaucoma. In retinal ganglion cells and astrocytes, mitochondrial dysfunction may be a factor in issues with high energy demand during metabolic stress brought on by elevated IOP. In those with normal IOP, glaucomatous optic neuropathy can also manifest, probably as a result of unusually low cerebrospinal fluid pressure in the subarachnoid space surrounding the optic nerve. The lamina cribrosa is subjected to a large pressure gradient as a result. Additional variables thought to play a role in glaucoma development include impaired microcirculation, altered immune responses, excitotoxicity, and oxidative stress. Neurodegeneration of retinal neurons and cells in the central visual pathway can result from primary degenerative processes in neural tissues that alter the environment and make them more susceptible to injury. These intricate pathways demonstrate how glaucoma is multifactorial and how several biological systems are involved in the aetiology of the condition. To completely comprehend these pathways and create efficient treatment approaches to stop or reduce the progression of glaucoma, more study is required [19].

Clinical presentation and diagnosis

**Figure 3:**
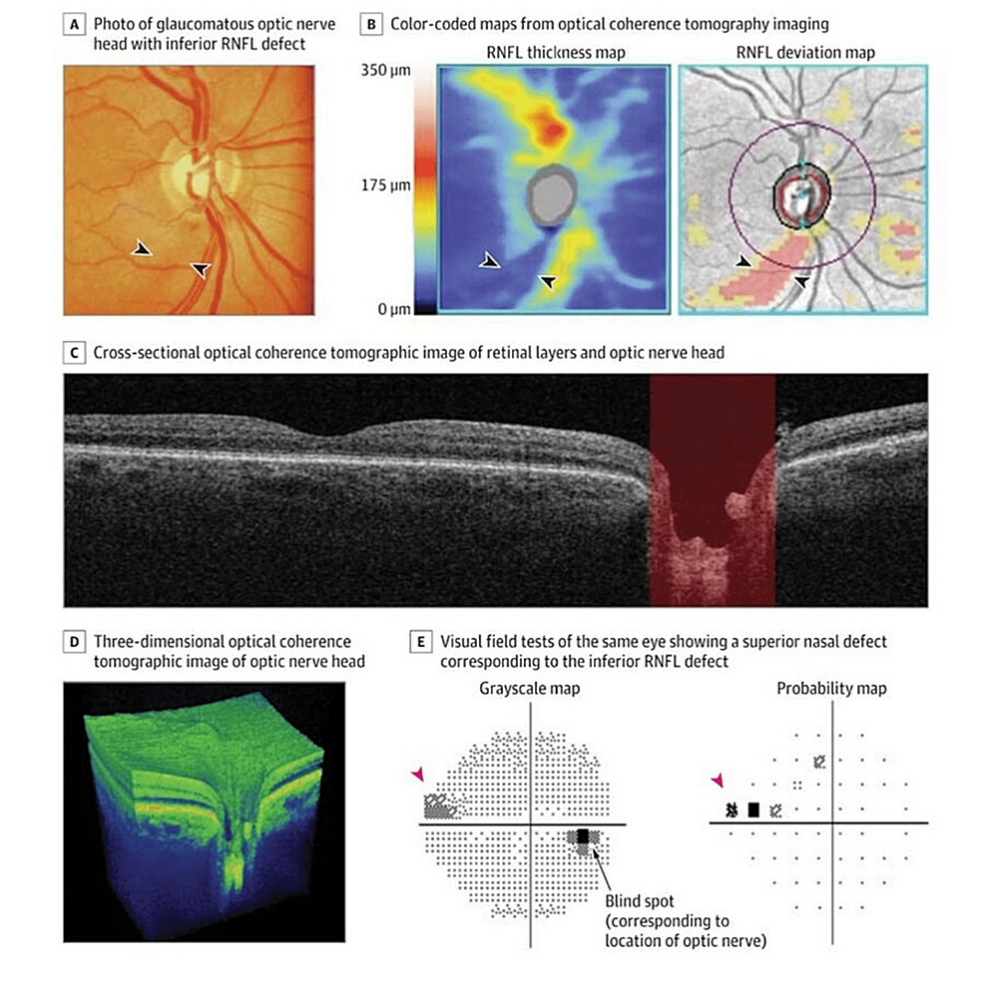
Imaging Assessment of the Optic Nerve and Retinal Nerve Fiber Layer Using Spectral Domain Optical Coherence Tomography A: The arrowheads point to a retinal nerve fiber layer (RNFL) defect. B: Areas of thicker RNFL appear in yellow and red. Arrowheads point to the RNFL defect. A deviation map compares the RNFL thickness values with a normative database and highlights the defect. E: Arrowheads point to a visual field defect [24-28]. [Source: JAMA Network Open]

Even though studies have shown that many glaucoma patients have IOP levels below 22 mmHg, high IOP is still a significant risk factor for the disease [20,21]. However, even with long-term follow-up, not everyone with increased IOP develops glaucoma [20]. Glaucoma frequently goes unnoticed until the optic nerve has suffered severe harm. Once symptoms appear, vision loss and a lower quality of life are possible, which emphasizes the value of early intervention. Referring people at glaucoma risk to an eye care professional is essential. An ophthalmoscopic examination of the optic nerve head can reveal changes in the optic nerve head and the retinal nerve fibre layer, which are crucial diagnostic indications of glaucoma. For early detection, a thorough ophthalmological examination is essential. Typically beginning in the midperiphery and moving inward, glaucoma causes the visual field to gradually deteriorate, giving rise to central or peripheral islands of vision. Due to the lack of a clear reference standard, early diagnosis might be difficult. Although the optic nerve head may be examined to look for evidence of neuronal death, the variability in appearance among the healthy population makes it difficult to identify damage early. Although regular testing may miss deficiencies until a large loss of retinal ganglion cells (30%-50%) has occurred, visual field impairments confirm the diagnosis [22,23]. The diagnosis of glaucoma depends on the longitudinal assessment of structural optic nerve damage. Optic nerve head photography and ophthalmoscopy are two techniques that can be used to accomplish this. However, it can be difficult to subjectively diagnose optic disc degeneration, which can result in large discrepancies among medical professionals. To get around this, cutting-edge imaging methods such as optical coherence tomography, scanning laser polarimetry, and confocal scanning laser ophthalmoscopy give factual, numerical information on the loss of optic nerve fibres. These imaging techniques aid in the early detection of the illness and the monitoring of its development over time (Figure 3) [24-28]. Identification of people with a family history of glaucoma and ensuring they receive a thorough ophthalmological assessment are critical functions of primary care physicians. People with a family history of blindness who have not had a dilated fundoscopy in the previous two years should be referred for further testing. Primary care physicians may detect optic nerve damage symptoms during routine clinical visits using direct ophthalmoscopy, which may require referring the patient to an ophthalmologist [29].

Genetic approaches to glaucoma research

Numerous techniques have been used to pinpoint the genetic components of glaucoma. Using families with numerous affected members, genetic linkage analysis has been extensively used to identify genes inherited as Mendelian traits [30]. The chromosomal region surrounding a linkage locus is next evaluated for potential disease-associated genetic variations. Whole exome sequencing, which entails sequencing the complete coding region of the genome, is now possible because of recent advancements in high-throughput DNA sequencing. Untranslated genomic regions are not included in whole exome sequencing, which is restricted to protein-coding regions. Whole exome sequencing leaves a minor coding region gap because of the sequence collecting method. However, this technique has been effective in locating genetic variants or causal mutations for uncommon Mendelian disorders, such Miller syndrome, congenital chloride-losing diarrhea, and Schinzel-Giedion syndrome [31]. This technology can be used to find uncommon causative variations, which is a potent method for improving our comprehension of particular disease mechanisms. Researchers frequently employ a variety of techniques, including admixture mapping and the genome-wide association study (GWAS), for both common and complicated illnesses. Single nucleotide polymorphisms (SNPs), among other genetic markers, are included in GWAS datasets that contain thousands of cases and controls. This technique is effective at locating common genetic variations linked to particular traits or disorders. GWAS results have shown disease-associated genetic variations in a variety of human diseases and characteristics, including age-related macular degeneration (AMD), POAG, and exfoliation glaucoma (XFG), even if the underlying mechanisms are still unknown. On the other hand, to find disease-associated polymorphisms, mixture mapping relies on variations in disease prevalence among groups. The localization of a trait to a particular genomic region is made possible by allele frequency changes in disease-causing genetic variants, which contribute to some of these discrepancies. This strategy is currently being used by researchers to look at the genetic causes of POAG among African-Americans. The impact of genetic imprinting and DNA copy number variations on hereditary illnesses is another topic of research. Together, these cutting-edge methods are making tremendous progress in our comprehension of glaucoma, particularly POAG [32].

Treatment

The major objectives of glaucoma treatment are to slow the course of the condition and preserve the patient's quality of life. Because the impact on quality of life might happen sooner than initially anticipated, early detection and management are essential. Glaucoma can only be effectively treated by lowering IOP or eye pressure [33]. Several multicenter clinical trials have shown that lowering IOP can aid in delaying the start and development of the condition. As an illustration, the OHT study targeted people with ocular hypertension, which is defined as a high IOP without glaucoma symptoms. In comparison to the untreated group (9.5%), the treatment group (after a 5-year follow-up) had a lower incidence of acquiring glaucoma symptoms (4.4%). Similar to this, participants in the early manifest glaucoma trial (EMGT) [34] were randomized to either therapy or no treatment for their identified glaucoma. After a median follow-up of six years, the group receiving treatment had a lower risk of illness progression (45%) than that of the control group (62%). These studies emphasize the value of early intervention and the significance of IOP lowering in the treatment of glaucoma. The risk of disease development can be reduced with effective IOP reduction, protecting the patient's eyesight and general well-being. For glaucoma patients, regular monitoring and specialized treatment regimens are essential to achieving the best results. The American Academy of Ophthalmology's current treatment recommendations advocate reducing IOP to a goal level that slows disease development enough to minimize functional impairment. The goal IOP is defined by parameters such as pre-treatment pressure levels linked with retinal damage, the severity of the damage, risk factors for progression, life expectancy, and probable treatment side effects. The primary goal is generally to reduce IOP by 20%-50%; however, this may need to be altered during follow-up based on illness development [35]. There are several types of pressure-lowering medicines available for the treatment of glaucoma. Because of their effectiveness and low systemic side effects, prostaglandin analogues are frequently used as first-line treatment. They function by lowering outflow resistance and enhancing aqueous humor flow through the uveoscleral route. Other types of topical medicines are less successful; however, they can be used as a last resort or when prostaglandin analogues are contraindicated or not tolerated. Patients must adhere to the treatment regimen, and efforts should be made to emphasize the necessity of adherence. Laser or incisional operations may be explored if medicinal therapy fails to produce enough IOP reduction or is associated with intolerable side effects. Laser trabeculoplasty is an outpatient department (OPD) procedure that causes alterations in the trabecular meshwork to promote aqueous outflow. The most frequent incisional surgical operation is trabeculectomy, which involves constructing a drainage path for aqueous humor. Alternative techniques, known as minimally invasive glaucoma operations, have been created and are being studied because they have a lower risk of sight-threatening complications than trabeculectomy. It is important to note that, despite efforts to create neuroprotective medications to prevent optic nerve injury, there is presently no substantial data confirming their efficacy in slowing disease progression in glaucoma patients. A lack of knowledge of the underlying processes, limited availability of medications targeting these systems, and a lack of a feasible regulatory approach for therapeutic approval are all challenges in this domain. Overall, glaucoma therapy focuses on decreasing IOP using medication, laser techniques, or surgery, with the objective of delaying disease progression and maintaining vision and quality of life [19].

## Conclusions

Glaucoma, a leading cause of blindness, results from optic nerve changes and the gradual loss of retinal ganglion cells. Early detection and improved diagnostics are vital due to underdiagnosis. Risk factors include age, gender, myopia, migraines, and conditions such as diabetes and smoking. Genetic studies, including GWAS and DNA analysis, have enhanced our understanding, aiding diagnosis. Imaging techniques such as optical coherence tomography provide objective data for early detection. Ongoing research and technology improve outcomes. Patients with family history should see an eye specialist. Glaucoma often coexists with age-related conditions. Treatment focuses on reducing intraocular pressure through drugs, laser treatments, or surgery. Adherence to treatment is crucial. Neuroprotective drugs for optic nerve preservation are under study. In summary, knowledge of glaucoma's risk factors, genetics, diagnostics, and treatment options is essential for early detection, effective management, and better outcomes. Staying informed and vigilant about eye health can significantly impact one's quality of life and vision preservation.
